# Supplementary material for: Multi‐dataset identification of innovative feature genes and molecular mechanisms in keratoconus
Source: J Cell Mol Med. 2024 Sep 19;28(18):e70079. doi: 10.1111/jcmm.70079 (PMC11412914; doi:10.1111/jcmm.70079)
Supplement: Supplementary file 2 — Material S2. [file JCMM-28-e70079-s002.zip › 7.GSEA analysis/4.MYRF/KEGG_PRION_DISEASES.html]

Details for gene set KEGG\_PRION\_DISEASES[GSEA]

|  || Dataset | MYRF.group.cls#h\_versus\_l |
| Phenotype | group.cls#h\_versus\_l |
| Upregulated in class | l |
| GeneSet | KEGG\_PRION\_DISEASES |
| Enrichment Score (ES) | -0.5337657 |
| Normalized Enrichment Score (NES) | -1.5274006 |
| Nominal p-value | 0.022222223 |
| FDR q-value | 0.21099144 |
| FWER p-Value | 0.72 |
Table: GSEA Results Summary

  

Fig 1: Enrichment plot: KEGG\_PRION\_DISEASES      
 Profile of the Running ES Score & Positions of GeneSet Members on the Rank Ordered List

  

| SYMBOL | TITLE | RANK IN GENE LIST | RANK METRIC SCORE | RUNNING ES | CORE ENRICHMENT || 1 | C9 | na | 807 | 0.496 | 0.0456 | No |
| 2 | C8B | na | 1870 | 0.368 | 0.0661 | No |
| 3 | ELK1 | na | 3767 | 0.262 | 0.0478 | No |
| 4 | C8A | na | 6904 | 0.167 | -0.0194 | No |
| 5 | C6 | na | 7632 | 0.148 | -0.0199 | No |
| 6 | PRKX | na | 11663 | 0.068 | -0.1266 | No |
| 7 | MAP2K2 | na | 14012 | 0.038 | -0.1890 | No |
| 8 | MAPK3 | na | 14382 | 0.034 | -0.1950 | No |
| 9 | C1QC | na | 16083 | 0.017 | -0.2416 | No |
| 10 | BAX | na | 17915 | 0.000 | -0.2943 | No |
| 11 | HSPA1A | na | 18580 | -0.006 | -0.3126 | No |
| 12 | PRKACA | na | 19584 | -0.017 | -0.3392 | No |
| 13 | MAPK1 | na | 20406 | -0.027 | -0.3592 | No |
| 14 | STIP1 | na | 20780 | -0.031 | -0.3656 | No |
| 15 | C7 | na | 21399 | -0.038 | -0.3781 | No |
| 16 | SOD1 | na | 21437 | -0.039 | -0.3738 | No |
| 17 | HSPA5 | na | 22668 | -0.055 | -0.4017 | No |
| 18 | NOTCH1 | na | 23798 | -0.072 | -0.4242 | No |
| 19 | C8G | na | 24861 | -0.091 | -0.4422 | No |
| 20 | EGR1 | na | 25048 | -0.094 | -0.4345 | No |
| 21 | PRNP | na | 25610 | -0.105 | -0.4361 | No |
| 22 | LAMC1 | na | 27992 | -0.160 | -0.4825 | No |
| 23 | C1QA | na | 29773 | -0.216 | -0.5037 | Yes |
| 24 | MAP2K1 | na | 30150 | -0.229 | -0.4827 | Yes |
| 25 | CCL5 | na | 31086 | -0.268 | -0.4724 | Yes |
| 26 | PRKACG | na | 31385 | -0.285 | -0.4414 | Yes |
| 27 | C5 | na | 32047 | -0.320 | -0.4160 | Yes |
| 28 | NCAM1 | na | 32848 | -0.376 | -0.3869 | Yes |
| 29 | IL1A | na | 32896 | -0.380 | -0.3354 | Yes |
| 30 | NCAM2 | na | 33101 | -0.397 | -0.2861 | Yes |
| 31 | IL6 | na | 33353 | -0.425 | -0.2343 | Yes |
| 32 | C1QB | na | 33420 | -0.433 | -0.1760 | Yes |
| 33 | PRKACB | na | 33438 | -0.435 | -0.1161 | Yes |
| 34 | FYN | na | 33971 | -0.517 | -0.0596 | Yes |
| 35 | IL1B | na | 34248 | -0.586 | 0.0138 | Yes |
Table: GSEA details [plain text format]

  

Fig 2: KEGG\_PRION\_DISEASES      
 Blue-Pink O' Gram in the Space of the Analyzed GeneSet

  

Fig 3: KEGG\_PRION\_DISEASES: Random ES distribution      
 Gene set null distribution of ES for **KEGG\_PRION\_DISEASES**
